# Fumigant Antifungal Activity of *Corymbia citriodora* and *Cymbopogon nardus* Essential Oils and Citronellal against Three Fungal Species

**DOI:** 10.1155/2014/492138

**Published:** 2014-01-30

**Authors:** Raimundo Wagner de S. Aguiar, Marcio A. Ootani, Sérgio Donizeti Ascencio, Talita P. S. Ferreira, Manoel M. dos Santos, Gil R. dos Santos

**Affiliations:** ^1^Universidade Federal do Tocantins, Campus Universitário de Gurupi, 77410-530 Gurupi, TO, Brazil; ^2^Universidade Federal do Tocantins, Campus Universitário de Palmas, 77.020-210 Palmas,TO, Brazil

## Abstract

*Corymbia citriodora* and *Cymbopogon nardus* essential oils samples were analyzed by GC and GC-MS and their qualitative and quantitative compositions established. The main component of essential oils of *C. citriodora* and *C. nardus* was citronellal, at 61.78% and 36.6%, respectively. The essential oils and citronellal were tested for their fumigant antifungal activity against *Pyricularia* (*Magnaporthe*) *grisea*, *Aspergillus* spp., and *Colletotrichum musae*. The minimum inhibitory concentration (MIC) ranged from 100 to 200 ppm for the essential oils and 25 to 50 mg*·*mL^−1^ for citronellal. The contact assay using the essential oils and citronellal showed growth inhibition of the three fungal species. However, a concentration of 1.47 mg*·*mL^−1^ only reduced the inhibition of *Aspergillus* growth to 90% at 14 days of exposure. For the fumigant assay, 0.05, 0.11, and 0.23 mg*·*mL^−1^ of essential oils and citronellal drastically affected growth of *P. grisea, Aspergillus* spp., and *C. musae*. Harmful effects on the sporulation and germination of the three fungi were seen, and there was complete inhibition at 0.15 mg*·*mL^−1^ with both oils and citronellal. This showed that the crude component of essential oils of *C. citriodora* and *C. nardus* markedly suppressed spore production, germination, and growth inhibition of *P. grisea*, *Aspergillus* spp., and *Colletotrichum musae*.

## 1. Introduction

Field crops and stored grain are susceptible to attack by different pathogenic fungal species, one of the most serious agents of disease in tropical plants worldwide. The rice blast fungus, *Pyricularia *(*Magnaporthe*)* grisea*, causes serious disease in a wide variety of grasses including rice, wheat, and barley [1]. Banana fruit, commonly grown in Brazil, is regularly affected by anthracnose caused by *Colletotrichum musae*, resulting in a significant loss in production [2]. On the other hand, fungi colonize stored grain in low numbers before harvest but develop rapidly in storage when conditions are suitable. Among these, *Aspergillus *species *A. flavus *and *A. niger *are particularly common and may occur prior to harvest, forming mycotoxins [3].

Synthetic chemicals such as fungicide preservatives have long been used to reduce losses [4]. However, the application of these chemicals has led to a number of environmental and health problems because of their residual toxicity, carcinogenicity, hormonal imbalance, and toxicity to sperm [5]. Moreover, because of indiscriminate use of fungicides, *P. grisea*, *Aspergillus* species, and *C. musae* fungi have developed resistance to the most widely used fungicides [6–8].

Plant essential oils may provide alternatives to currently used fungicidal agents because of their contact inhibition and volatile effect. They constitute a rich source of control of different fungal species and can exert strong antifungal activities [9, 10]. Studies using *Corymbia citriodora *and *Cymbopogon nardus* essential oils have revealed broad-spectrum activities against various fungal species [11]. In some cases, activities have been associated with the major constituent, citronellal, providing potential antimicrobial agents [12]. It is difficult to correlate the activity to single compounds or classes of compounds to different fungi species. It seems that antifungal and antimicrobial effects are the result of many compounds acting synergistically.

On the other hand, antifungal activities have not been associated with the concentrations of citronellal in *C. citriodora *and *C. nardus* essential oils. However, different concentrations and compositions have been reported for citronellal in* C. citriodora *and *C. nardus* essential oils [13]. Citronellal activities have not yet been investigated for *P. grisea* and *C. musae*, but citronellal is reported to show activities against fungi from *Aspergillus* [11]. Thus, there would be a negligible chance of development of resistant races of fungi after application of essential oils to fruit and after harvest. Consequently, novel, broad-spectrum, nontoxic antifungal compounds, appropriate for empirical use and not prone to selection of resistant organisms, are required.

The objective of this study was to test and compare the antifungal activities of *C. citriodora, C. nardus *essential oils and citronellal constituent isolated against *P. grisea*, *Aspergillus* spp., and *C. musae *and evaluate the potential application to control phytopathogenic fungi.

## 2. Material and Methods

### 2.1. Plant Material and Steam Distillation

*C. nardus* and *C. citriodora* were collected in Gurupi (11°43′45′′ latitude S, 49°04′07′′ Longitude W) and Dueré (11°20′38′′ latitude S, 49°16′14′′ W Longitude W), Tocantins, Brazil. Taxonomic identification was confirmed by experts at the herbarium (Federal University of Tocantins, Brazil), where samples were deposited with reference numbers 10.741 and 10.742. *Cymbopogon nardus* and *Corymbia citriodora* essential oils were extracted from the leaves by steam distillation method in a Clevenger apparatus, as described by [14]. Citronellal compound was purchased from Sigma-Aldrich (St. Louis, MO, USA) and stored at 4°C until further analysis, before antifungal experiments were conducted.

### 2.2. Fungus Strains

Fungi were isolated from *Oryza sativa* plants (*P. grisea*), stored grain (*Aspergillus *spp.), and banana fruits (*C. musae*). Their identity was confirmed based on colony morphology and spore characteristics according to Kimati et al. [15]. Single spore cultures of these fungus strains were maintained on PDA (potato dextrose agar) medium at 27°C. Inoculum was prepared by using the conidia of two-week-old cultures of the fungi. The conidia were dislodged from the surface of the medium by flooding with sterile distilled water and gentle rubbing with a sterile glass rod. The suspensions were filtered through cotton wool to remove mycelial fragments and adjusted to 10^5^ conidia/mL. Following this, all three conidial and spore suspensions were used for inoculation in PDA culture medium.

### 2.3. Gas Chromatography-Mass Spectrometry (GC-MS) Analysis

The GC analysis of *C. nardus* and *C. citriodora *essential oils was performed using a Chemito 8510 GC instrument equipped with a data processor. A BP-5 wide-bore capillary column (30 m–0.53 mm i.d., 1.0 mm film thickness) was used for separation of the sample components (sample size 0.03 mL, measured using a Hamilton GC syringe with 1.0 mL cap.). Hydrogen was used as the carrier gas at a flow rate of 5 mL/min and 20 p.s.i. inlet pressure. The GC column oven temperature went from 70°C to 210°C at a rate of 2.5°C/min, with a final hold time of 5 min. Both injector and detector (FID) temperatures were maintained at 230°C. GC-MS analysis was carried out on a Trace DSQ MS (Thermo Electron Corporation), using a BP-5 capillary column (30 m × 0.25 mm × 0.25 *μ*m), with helium as the carrier gas at a flow rate of 1 mL/min; split ratio 1 : 20. The column temperature went from 65°C to 210°C (10 min hold) at 3°C/min. Mass spectra were recorded in the range 40–650 amu, operating at 70 eV, and the ion source temperature was maintained at 200°C. The constituents of the oil were identified using standard reference compounds and by matching the mass spectra fragmentation pattern with NIST Mass Spectra Library stored in the GC-MS database.

### 2.4. Antifungal Assays

#### 2.4.1. Minimum Inhibitory Concentration (MIC)

The minimum inhibitory concentration of *C. nardus* and *C. citriodora *essential oils and citronellal was established by carrying out the twofold dilution method against *Aspergillus *spp., *P. grisea*, and *C. musae* Adjou et al. [16]. The solutions were serially diluted with PDA in water to the final concentration of *C. citriodora *and *C. nardus *(0.5, 1.0, 1.5, 25, 50, 100, 200, 300, 400, and 500 ppm) and citronellal (0.5, 1.0, 1.5, 25, 50, 100, 150, 200, and 250 ppm). A 10 *μ*L spore suspension of each test fungus strain was inoculated in the test tubes in PDA and incubated for 7 days at 27°C. The control tubes containing PDA were inoculated with only fungal suspension. The minimum concentration at which no visible growth was observed was defined as the MIC, expressed in ppm.

#### 2.4.2. Contact Assay

The effect of *C. nardus* and *C. citriodora *essential oils and citronellal on the growth of *Aspergillus *spp., *P. grisea*, and *C. musae *in PDA at 27°C was studied according to the poisoned food technique (PF) of Grover and Moore [17]. Sterilized Petri dishes were filled with 20 mL of PDA and oil was added to get the required concentrations 0.10, 0.13, 0.15, 0.19, 0.47, 0.63, 0.94, 1.26, and 1.57 mg·mL^−1^ for essential oils of *C. nardus* and* C. citriodora *and citronellal on top of the PDA medium. In the medium, 0.05 mL/100 mL Tween-80 was added for even distribution of the oils. The test fungi were inoculated with 5 mm mycelial plugs from 14-day-old cultures and incubated at 25 ± 2°C. The growth of fungal species was recorded after two weeks of incubation. Growth inhibition of treatment against control was calculated by Hossain et al. [18], using the following formula:

(1) Inhibition  ratio  (%) =1−mycelium  growth  treatment  (cm)mycelium  growth  control  (cm)×100.

#### 2.4.3. Volatile Assay

The effect of *C. nardus* and *C. citriodora *essential oils and citronellal on the growth of *Aspergillus *spp., *P. grisea*, and *C. musae *in PDA at 27°C was studied according to the technique of Grover and Moore [17]. The Petri dishes were inverted and sterile filter paper discs (4 mm diameter) were impregnated with *C. nardus *and* C. citriodora *essential oils and citronellal (0.007, 0.01, 0.02, 0.03, 0.1, 0.15, 0.17, 0.23, and 0.25 mg·mL^−1^). Distilled water with 5% Tween was attached to the inverted lid (1 disc/Petri dish). The Petri dishes were then wrapped with Parafilm along the rim to check for the release of the volatile components, inverted, and incubated for 14 days at 27 ± 2°C. The radial growth of the mycelium was recorded and results were expressed as percentage of fungal colony growth by the formula described by [18].

#### 2.4.4. Spore and Germination Inhibition Assay

Five concentrations of oil (0.03, 0.06, 0.09, 0.12, and 0.15 mg·mL^−1^) and two controls (one with sterile distilled water and the other with Tween 80% (0.03%) in sterile distilled water) were tested for spore product and germination inhibition of *Aspergillus *spp., *P. grisea*, and *C. musae *from volatile *C. nardus* and *C. citriodora *essential oils and citronellal. Aliquots of 0.1 mL from each were mixed with fungal spores obtained from 14-day-old cultures of the fungi and placed on separate glass slides in triplicate. Slides containing the spores were incubated in a moist chamber at 27°C for 14 days. Spore germination and inhibition were quantified according to Rana et al. [19]. About 200 spores were counted and the percentages of spore germination and inhibition were calculated.

### 2.5. Statistical Analysis

All experiments were repeated four times. Significant differences between values were determined by using Duncan's multiple range test (*P* < 0.05), following one-way ANOVA. Statistical analysis was performed using SISVAR 4.6 Ferreira [20] and graphs were produced using SIGMA PLOT 11.0.

## 3. Results and Discussion

*C. citriodora *and *C. nardus* essential oils samples were analyzed by GC and GC-MS and their qualitative and quantitative compositions were established (Table 1). The main component of essential oils of *C. citriodora *and *C. nardus* was citronellal, with values of 61.78% and 36.6%, respectively. Substantial amounts of geraniol, *β*-citronellol, and isopulegol were also found. The observed concentration of the main constituents in the essential oils of *C. nardus* and *C. citriodora *was different from that reported by Nakahara et al. [11] and Olivero-Verbel et al. [13]. These changes in the compositions of essential oils might arise from several environmental (climatic, seasonal, or geographic) and genetic differences [21–23].

The minimal inhibitory concentration (MIC) of the essential oils of *C. nardus *and* C. citriodora *and citronellal is shown in Table 2. The MIC values indicate that the essential oil of *C. nardus* was more efficient than that of *C. citriodora, *totally inhibiting fungal growth (Table 2). The lowest MIC values found for citronellal demonstrate that the fungal species used in this study are susceptible to the compound, with MIC values ranging from 50 to 25 ppm (Table 2).

The inhibitory effects against fungi at concentration 0.47 mg·mL^−1^ are shown in Table 3. The citronellal constituent had a good inhibitory effect on *Aspergillus *spp., and *C. musae *inhibited the radial growth completely, while for *C. musae *similar results were not observed. These results suggested that citronellal contributed significantly to antifungal activity of *C. nardus *and* C. citriodora*. There have been studies supporting these results, especially showing that the active constituent citronellal was a potent inhibitor (via vapor phase) of various fungi at ambient temperatures [11].

It should also be considered that this activity of *C. nardus *and* C. citriodora* essential oils may be associated with synergistic interactions among the essential oil components. There have been studies supporting these results, showing that the strong antifungal activity of *C. nardus* essential oils associated with the effect of synergism occurred between citronellal and linalool, as reported by [11].

**Figure 1:**
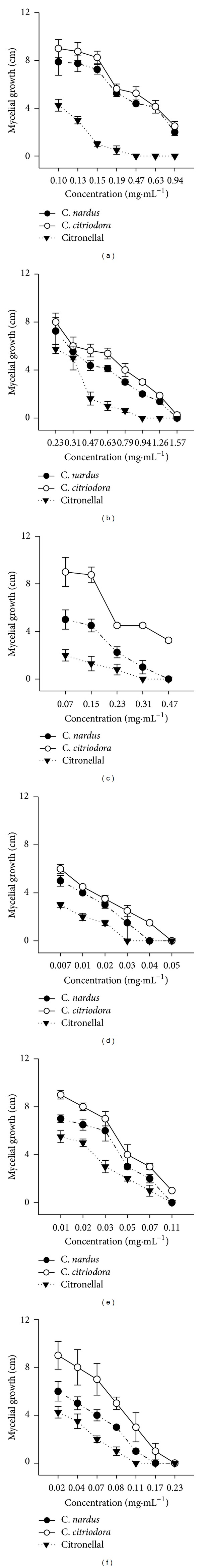
Effects of essential oils of *C. nardus*, *C. citriodora*, and the major constituent citronellal in PDA on the mycelial development of *P. grisea* (a), *Aspergillus *spp. (b), and *C. musae* (c). Volatile effects of *C. nardus*, *C. citriodora,* and citronellal on mycelial development of *P. grisea* (d), *Aspergillus* spp. (e), and *C. musae* (f), 14 days after treatment.

The results, presented in Figures 1(a), 1(b), and 1(c), reveal that essential oils of both* C. nardus* and *C. citriodora *and citronellal all have a strong effect on the growth of *P. grisea, Aspergillus *spp., and *C. musae*. This antifungal activity is comparable to that reported by [22, 24]. The stronger activity of the essential oil of *C. nardus *and* C. citriodora *against fungal species may also be because of the antifungal properties of citronellal. This is in agreement with what was observed by [25], who observed that citronellal has a strong effect on the growth of *Aspergillus niger*. However, other compounds, such as geraniol, *α*-pinene, *β*-pinene, myrcene, and linalol, as well as lower concentrations of the essential oils of *C. nardus and C. citriodora*, have antifungal activity [11].

The fumigant activity of *C. nardus *and *C. citriodora *essential oils and citronellal inhibited fungal strains (Figures 1(d), 1(e), and 1(f)). Fumigant activity of *C. nardus* essential oil was more significant, and it is probable that the antifungal volatile activity of essential oils is concentration-dependent. Nevertheless, the citronellal results are similar to those obtained by [11], suggesting that constituents contribute significantly to fumigant activity of *C. nardus C. citriodora *essential oils.

**Figure 2:**
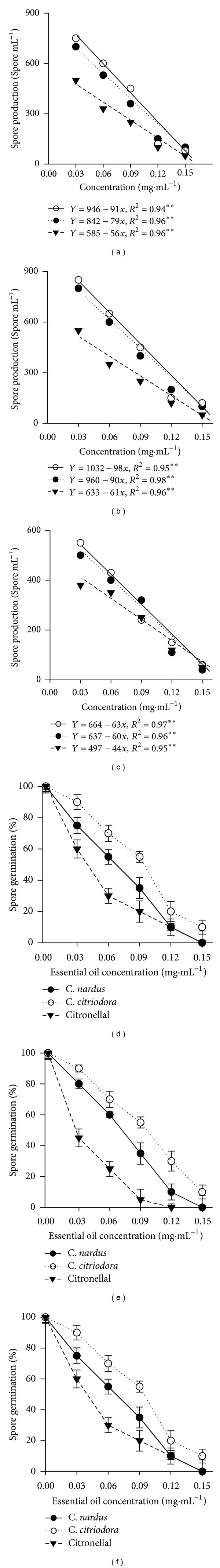
Effects of volatile compounds of *C. nardus*, *C. citriodora, *and the major constituent citronellal in the sporulation of *P. grisea* (a), *Aspergillus* spp. (b), and* C. musae *(c). Volatile effects of *C. nardus*, *C. citriodora*, and citronellal on spore germination (%) of *P. grisea* (d), *Aspergillus* spp. (e), and *C. musae* (f), 14 days after treatment.

The spore production of *P. grisea*, *Aspergillus *spp., and *C. musae* was drastically affected by volatile activity of *C. nardus* and *C. citriodora *essential oils and citronellal, with a greater inhibition of spore production (Figures 2(a), 2(b), and 2(c)). The analyses revealed spore production of all fungi to be significantly affected (*P* < 0.05) by the oils, and this was inhibited completely at 0.15 mg·mL^−1^ (Figures 2(a), 2(b), and 2(c)). It is therefore likely that suppression of spore production by essential oil treatment could make a major contribution to limiting the spread of the pathogen by lowering the spore load in the storage atmosphere and on surfaces. This report describes the complex effect of essential oils from* C. nardus* and *C. citriodora *and citronellal on spore germination, with a result comparable to that obtained with other essential oils on different fungal species [26, 27].

Antifungal activity of volatile essential oils against various fungal species has been reported, including *Colletotrichum coccodes* in apple (*Malus pumila* L.) and tomato (*Lycopersicon esculentum *Mill.) and *Lasiodiplodia theobromae* (Pat.) Griffon and Maubl, *C. musae* Berk. and Curtis, and *Fusarium* spp. in banana [28–30]. Therefore, according to the results of this study, antifungal activity is associated with the citronellal constituent concentration of essential oils present in both plant species in this study, with potential applications in controlling plant pathogenic fungi that cause severe destruction in crops.

In this study, *C. nardus* and *C. citriodora *essential oils' antifungal action could be attributed to the presence of phenolic compounds, oxygenated monoterpenes, and sesquiterpene hydrocarbons [31]. In our opinion, as a major component of *C. nardus* and *C. citriodora *essential oils, citronellal plays a key role in their antifungal activities. The antifungal activity of the compounds has been reported by others [25, 32]. On the other hand, the components, at a lower concentration, also contributed to the antifungal activity of the oils [33]. Therefore, it would also be interesting to study the effects of essential oils as new antifungal agents to control *P. grisea*, *Aspergillus* spp., and *C. musae*, reducing postharvest and stored grain losses.

In general, the antifungal activity of *C. nardus* and *C. citriodora *essential oils and citronellal against *C. musae, P. grisea*, and *Aspergillus *spp. can be attributed to morphological changes in the cell wall and interference in enzymatic reactions of wall synthesis, which affect fungal growth and morphogenesis [34]. This causes either an increase in ion permeability and leakage of vital intracellular constituents, or impairment of the fungal enzyme systems [35, 36].

This study indicated that essential oils may possess antifungal activity and can be exploited as an ideal treatment for future plant disease management programs, eliminating fungal spread. The mechanism underlying the action of essential oil-enrichment on the switch between vegetative and reproductive phases of fungal development is still unclear. The impact of *C. nardus* and *C. citriodora *essential oils on sporulation may reflect effects of the volatiles emitted by oils on surface mycelial development and the perception and transduction of signals involved in the switch from vegetative to reproductive development.

## Figures and Tables

**Table 1:** Chemical composition, concentrations (%), and Kovats indices (calculated and tabled) for essential oils of *Cymbopogon nardus *and* Corymbia citriodora. *

Chemical compound	Concentrations (%)		Kovats indices	
	*Cymbopogon nardus *	*Corymbia citriodora *	IKtab	IKcalc
*β*-Pinene	—	2.83	980	980
1,8-Cineol	—	3.44	1033	1027
Isopulegol	1.40	15.54	1146	1144
Citronellal	36.53	61.78	1153	1152
*β*-Citronellol	13.10	7.90	1228	1229
(Z)-Caryophyllene	—	2.13	1404	1399
Linalool	1.1	—	1094	1098
Geraniol	25.56	—	1255	1249
Citronellyl acetate	2.22	—	1354	1351
Geraniol acetate	1.51	—	1383	1386
Germacrene-D	0.69	—	1480	1489
Delta cadinene	1.09	—	1524	1516
Elemol	8.24	—	1549	1548
Germecrene-D-4-ol	1.64	—	1574	1573

Total	91.98	93.62	—	—

**Table 2:** Screening of (ppm) of *Cymbopogon nardus *and* Corymbia citriodora* and citronellal essential oils by the PF technique.

Essential oils	Fungi		
	*Aspergillus* spp.	*Pyricularia grisea *	*Colletotrichum musae *
	MIC	MIC	MIC
*C. nardus *	100	100	50
*C. citriodora *	200	200	50
Citronellal	25	50	25

The values are the average of three determinations. Minimal inhibitory concentration (MIC).

**Table 3:** Percent of inhibition (%) of essential oils and the major constituent citronellal (0.47 mg·mL^−1^) in PDA in the micelia development of *P. grisea*, *Aspergillus *spp., and *C. musae*, after 14 days.

Essential oils	Fungi		
	*P. grisea *	*Aspergillus *spp.	*C. musae *
*C. nardus *	61 ± 1.1^b^	57 ± 2.4^b^	100 ± 2.9^a^
*C. citriodora *	65± 1.4^b^	66 ± 1.9^b^	95 ± 3.2^a^
Citronellal	55 ± 1.0^b^	100 ± 1.3^a^	100 ± 2.6^a^

^a^Values are the mean of three replicates ± SE. Where the letters are the same, there is no significant difference between the fungal species at this concentration (0.47 mg·mL^−1^).
